# Genetic and laboratory diagnostic approach in Niemann Pick disease type C

**DOI:** 10.1007/s00415-014-7386-8

**Published:** 2014-08-22

**Authors:** K. McKay Bounford, P. Gissen

**Affiliations:** 1 West Midlands Regional Genetics Service, Birmingham Women’s Hospital, Birmingham, UK; 2 University of Birmingham, Birmingham, UK; 3 UCL Institute of Child Health, London, UK; 4 MRC Laboratory for Molecular Cell Biology, University College London, London, UK; 5 Paediatric Metabolic Medicine, Great Ormond Street Hospital for Children, Great Ormond Street, London, WC1N 3JH UK

**Keywords:** Niemann Pick type C disease (NP-C), Diagnosis, Genetics, *NPC1*, *NPC2*

## Abstract

Niemann Pick disease type C (NP-C) is a rare autosomal recessive disorder that results from mutations in either the *NPC1* or the *NPC2* gene. The estimated incidence of NP-C is 1 in 120,000 live births, although the frequency of cases is higher in some isolated populations. More than 350 different *NPC1* and *NPC2* gene mutations have been reported in patients with confirmed diagnoses. Approximately 95 % of patients harbour mutations in *NPC1*, with most of the remaining patients having *NPC2* mutations. The traditional methods for diagnosing patients with NP-C include histopathological analysis of bone marrow aspirate, liver and skin biopsies, fluorescent and electron microscopy, and cholesterol esterification assays. New laboratory methods that use mass spectroscopy for detection of cholesterol metabolism products are promising to become part of the routine diagnostic and screening tests in the near future, but further evaluation is required to determine the sensitivity and specificity of these analyses in patients with different age-at-onset forms of NP-C. Although filipin staining and cholesterol esterification studies performed in patient skin fibroblasts can, in experienced hands, provide a robust approach to diagnosing NP-C, they are only available in a few specialist laboratories. Thus, sequencing of *NPC1* and *NPC2* is currently the most universally accessible diagnostic technique in this disorder.

## Introduction

Niemann Pick disease type C (NP-C; OMIM#257220 and OMIM#607625) is a rare autosomal recessive, multisystemic, neurodegenerative condition caused by mutations in the *NPC1* or the *NPC2* gene. NP-C is also described as a neurovisceral disorder, as it affects visceral organs such as liver, spleen and lungs as well as the central and the peripheral nervous systems [28].

Clinical presentation varies greatly between patients in terms of age-at-onset and the type/severity of clinical symptoms, as well as in the speed of neurodegeneration. The incidence of NP-C is estimated to be 1:100,000–120,000 live births in France, the UK and Germany. However, studies in certain isolated populations have revealed a higher carrier frequency, as reported among Acadiens in Nova Scotia, Hispanics from Southern Colorado and New Mexico, and Greeks on a small Aegean island [21, 24, 38, 42]. The greater carrier frequency in these populations led to increased numbers of patients being detected.

The cellular pathology of NP-C is characterised by abnormal intracellular trafficking of cholesterol and other related lipids, with accumulation of unesterified cholesterol and other lipid moieties in lysosomes and late endosomes [29, 30, 36]. While the precise functions of the protein products encoded by the *NPC1* and *NPC2* genes have not yet been determined, it is believed that they may function in a co-operative fashion in the lysosomal/late-endosomal trafficking of cholesterol and other molecules [17, 35].

Other reviews in this supplement address the clinical features of NP-C in the context of current international disease management guidelines. In this review, we focus on the genetic and laboratory diagnostic approaches that can be applied when NP-C is suspected. The appropriate use of clinical and laboratory diagnostic methods addressed here will help to identify and treat patients early in the course of the disease.

## Genetics of NP-C

Carstea et al. [6] located *NPC1* on chromosome 18q11-q12 and first identified mutations in *NPC1* in patients with NP-C. The *NPC1* gene encodes a 1278 amino acid protein that belongs to a family of membrane-bound proteins with sterol-sensing domains. The complete *NPC1* genomic sequence consists of 57,052 kb [3]. The C-terminal section of NPC1 protein has sequence similarity to the Patched morphogen receptor. Topologic studies showed that it has 13 transmembrane domains and three hydrophilic luminal loops [12]. It was found that in monkey brain, NPC1 predominantly localises to late endosomes in presynaptic astrocytic glial processes [27]. Later studies concluded that neuropathology and cholesterol accumulation in NP-C is related to a defect in late-endosomal trafficking [45]. The current understanding of the intracellular cholesterol trafficking suggests that endocytosed cholesterol needs to be transported from late endosomes to the Golgi apparatus and then to the endoplasmic reticulum (ER) for esterification, which is essential for cholesterol secretion [8]. In NP-C cells, where NPC1 function is deficient, unesterified cholesterol and sphingolipids accumulate in late endosomes and lysosomes.

Lloyd-Evans et al. [18] reported increased storage of sphingosine and decreased lysosomal calcium levels in cell models of NP-C, suggesting that NPC1 may be involved in the lysosomal efflux of sphingosine and that its deficiency leads to changes in intracellular calcium concentration and a subsequent endocytic trafficking defect.

More than 300 mutations in *NPC1* have been reported so far, as listed across several mutation databases. There are also many mutations that were identified in service laboratories that have not been reported in the literature. The most up-to-date database, the Human Gene Mutation Database (www.hgmd.cf.ac.uk), lists a total of 334 *NPC1* defects that include 228 missense and nonsense mutations: 46 small deletions, 26 mutations affecting splicing, 24 small insertions, two small indels, seven gross deletions and one gross insertion/duplication. This list includes mutations located in the intronic sequence, which create additional splice sites [19].

The most common *NPC1* mutation, p.I1061T, is detected in ~20 % of NPC alleles among Western Europeans and is very common in Spanish–American patients from the upper Rio Grande valley [9, 22, 32]. The mutation p.G992W is found in the Nova-Scotian cohort of patients that, prior to the identification of the genetic cause of NP-C, were thought to have a unique disorder called Niemann Pick disease type D [11]. This mutation has since been found in patients with other ethnic origins. The region between amino acids 1038 and 1253, which shares 35 % identity with the patched gene, appears to be a hotspot for mutations [26]. Because of the large number of ‘private’ family-specific mutations, it is challenging to perform genotype–phenotype correlation studies. However, the presence of nonsense and frameshift mutations correlates well with the severe neurologic phenotype. Some of the missense mutations appear to be associated with late-onset, slowly progressing disease [22, 34].

Mutations in *NPC1* are found in ~95 % of patients with NP-C that underwent mutation testing, whereas only a few families with *NPC2* mutations have been reported so far. The *NPC2* gene is located on chromosome 14q24.3, spans 13.5 Kb, and contains five exons [25]. To date, one nonsense mutation (E20X) seems to occur more frequently in published reports [23, 41]. It is difficult to establish any genotype–phenotype correlation with such a small number of published reports [7].

The *NPC2* gene encodes a small 151 amino acid glycoprotein that is ubiquitously expressed and has a lysosomal and late-endosomal location [25]. Friedland et al. [10] determined the crystal structure of NPC2 protein and showed that it is able to bind the cholesterol analogue, dehydroergosterol. Further studies showed that NPC2 is a secreted, soluble protein with a high affinity for cholesterol [16]: a mutation of one amino acid codon in the *NPC2* gene led to the severe cholesterol trafficking defect that is characteristic of NP-C. The phenotype of *Npc2* knockout mice was found to be identical to that of the *Npc1* knockout, thus confirming that the two proteins are involved in the same intracellular pathway [35]. This neurodegenerative phenotype, characterised by excess lipid storage, was not enhanced in a double *Npc1* and *Npc2* knockout. Twenty disease-causing mutations have been reported in patients with *NPC2* mutations so far (www.hgmd.cf.ac.uk) including 15 missense/nonsense mutations, two mutations affecting splicing and three small deletions.

The onset and progression of neurological manifestations often correlate with specific mutations in both *NPC1* and *NPC2* genes, but this has not been found to be true in the case of visceral symptoms [39].

## Genetic testing for *NPC1* and *NPC2* mutations

The most frequently employed method of genetic analysis for NP-C is Sanger sequencing, which utilizes polymerase chain reaction (PCR) to target the 30 coding exons, and intron-exon boundaries, of the *NPC1* and *NPC2* genes. Alternatively, targeted next-generation sequencing (NGS) methods may be applied. NGS has the potential to reduce costs associated with genetic testing for NP-C, particularly if the genes concerned are included on a multi-gene panel along with other genes considered relevant to the patient phenotype. Multiple recent reviews describe various approaches to gene panel NGS [5, 14].

In general, sequence changes that result in the introduction of premature stop codons (i.e. nonsense, frameshift and conserved splice-site mutations) produce truncated mRNA species that are usually targeted for breakdown (nonsense-mediated decay; NMD) rather than translation into proteins. Sequence changes that result in small changes to the protein sequence (missense and in-frame mutations) may also be disease-causing and, as mentioned above, many of these have been associated with NP-C and are listed in mutation databases (www.hgmd.cf.ac.uk; http://npc.fzk.de). Lastly, sequencing may identify intronic sequence variants that can disrupt RNA splicing and lead to NMD. However, missense and intronic variants are not necessarily disease-causing, and several such polymorphisms exist in *NPC1* and *NPC2*.

The genetic diagnosis of NP-C requires the identification of clearly pathogenic mutations, but many families have ‘private’ sequence variants that have not been reported/published. If these sequence variants are missense or intronic changes, in silico protein and splicing prediction tools may be employed to assist in assigning their pathogenicity. Correlation of sequencing results with a patient’s clinical features and other diagnostic test results is useful for the interpretation of these cases.

Genomic rearrangements of *NPC1*, such as exonic deletions or whole-gene deletions, are a rare cause of the disease. Testing for this type of mutation requires a quantitative methodology and is most frequently performed using multiplex ligation-dependent probe amplification (MLPA, MRCHolland).

Deep intronic sequence variants, and variants in the promoter regions of *NPC1* and *NPC2*, are likely to be rare causes of NP-C. However, such defects would not be detected by the above methods. Full gene sequencing is possible using NGS methods, but this is likely to identify sequence variants that are difficult to interpret. Therefore, this strategy should be limited to patients with a clear clinical and biochemical diagnosis where no mutations have been identified using routine sequencing. Investigations of such sequence variants would require RNA and protein work and, as such, are often outside the scope of service laboratories.

## Other diagnostic procedures

The published international guidelines on the laboratory diagnosis of NP-C provide a diagnostic algorithm that proposes the use of the ‘filipin test’ on skin fibroblast cultures as the first-line specific investigation in patients with a strong suspicion of NP-C [28, 43]. The guidelines also suggest that clinical and laboratory features that should raise a strong suspicion of NP-C include: (1) abnormal bone marrow aspirate that in some cases may demonstrate filipin-positive foam cells; (2) elevated plasma chitotriosidase activities (moderately raised in most cases); and (3) the presence of (hepato)splenomegaly with normal activities of acid sphingomyelinase (excluding Niemann Pick type A and B diseases) and β-glucocerebrosidase (excluding Gaucher disease). After a positive or equivocal filipin test, the guidelines suggest *NPC1* and *NPC2* gene sequencing, initially testing the common mutation if relevant for the ethnic group in question.

### Bone marrow aspirate analysis

Foam cells and sea-blue histiocytes can be found in bone marrow from NP-C patients. Foam cells show high levels of unesterified cholesterol when stained with filipin. However, this test does not have good sensitivity as the features are not always identified. In addition, patients may need to undergo repeat bone marrow aspirations before a diagnostic appearance is detected.

### Electron microscopy of skin and liver biopsies

Electron microscopy findings on skin and liver biopsies can be supportive of the diagnosis of NP-C if vacuolation or hepatocytes containing ‘myelin figures’ are found [4, 15]. These features are representatives of the accumulated lipids due to the endo-lysosomal trafficking defect. Nevertheless, the specificity and sensitivity of these tests are low.

### Chitotriosidase assay

The chitotriosidase assay has been proposed as a screening test for NP-C. However, while it is mildly positive in some cases, it can also be normal. Ten per cent of the population have a pseudodeficiency mutation, which makes this test uninterpretable [33].

### Filipin test

The demonstration of impaired intracellular cholesterol transport and homeostasis is considered the most specific functional diagnostic test for NP-C. This test uses skin fibroblasts cultured in LDL-enriched medium and is able to detect unesterified cholesterol upon staining with filipin––a fluorescent chemical compound isolated from an actinomycete, *Streptomyces filipinensis*. Fluorescence microscopy of cultured skin fibroblasts from patients with NP-C usually demonstrates an increased number of vesicles staining positively with filipin. This appearance is described as the ‘classical’ storage pattern and can be identified in ~80–85 % of cases [38].

A smaller proportion of NP-C patients are found to have less obvious filipin staining, and are described as having a ‘variant’ biochemical phenotype. As discussed above, several recurrent *NPC1* mutations are known to be associated with this ‘variant’ phenotype [2, 40]. Heterozygotes can also show this pattern of cholesterol storage. Notably, such a variant phenotype, suggestive of ‘possible NP-C’, can also be seen in patients with sphingomyelinase deficiency (NP-A or NP-B), and other gene defects [2, 40].

Further biochemical testing can be undertaken to measure the LDL-induced rate of cholesteryl ester formation [40]. This test detects very low levels of cholesterol esterification in cell lines with a ‘classical’ biochemical phenotype, but only a mild or non-significant impairment is visible in those with a ‘variant’ phenotype [2, 40]. If performed in specialist laboratories with a lot of experience, these tests have high sensitivity and specificity. However, it is important to emphasise that ‘variant’ and ‘classical’ appearance on the filipin test do not correlate with clinical disease severity in NP-C.

### Development of new approaches to screening and diagnosis

Porter et al., looked for evidence of the accumulation of plasma cholesterol oxidation products (oxysterols) in NP-C patients, based on observations that oxysterol levels increase due to raised oxidative stress in multiple tissues in the mouse model of Npc1 deficiency [37, 44]. These investigators measured the concentration of cholestane-3β,5α,6β-triol (3β,5α,6β-triol) and 7-ketocholesterol (7-KC) in patients with *NPC1* mutations, and found that, in particular, 3β,5α,6β-triol was increased in the plasma of NP-C patients compared with controls [31]. The same research group developed a sensitive and specific method for oxysterol detection using liquid chromatography/tandem mass spectroscopy (LC–MS/MS), and measured 3β,5α,6β-triol and 7-KC in 109 patients with NP-C, 45 obligate carriers and 89 controls [13]. The mean plasma concentration of 3β,5α,6β-triol in NP-C patients was more than 5 times higher than in control subjects. However, there was overlap between measurements from NP-C patients and those from heterozygote *NPC1* mutation carriers [13]. Mean plasma concentration of 7-KC from NPC1 patients was more than six times higher than the controls, but again there was overlap with heterozygote carriers. The authors concluded that although oxysterol assay-based screening would have a positive predictive value of >97 % in an NP-C enriched population such as infants with cholestatic liver disease, this value would fall significantly if the tests were to be used in the general population.

Alvelius et al. [1] identified very high urine concentration of a C24 3b-sulfooxy-7b-*N*-acetylglucosaminyl-5-cholen-24-oic bile acid (SNAG-∆^5^-CA) together with its glycine and taurine-conjugates (SNAG-∆^5^-CG, SNAG-∆^5^-CT) in patients with NP-C compared with controls. As bile acids can be detected in urine and blood using the LC–MS/MS approach, the method for the detection of SNAG-∆^5^-CA in urine was developed and tested by comparing samples from one adult-onset and one childhood-onset NP-C patient with 11 controls (including two patients with the bile-acid synthesis disorder, 3b-HSD deficiency) [20]. The authors found that SNAG-∆^5^-CA and its amides were 400 times greater in the infant NP-C patient and 40 times greater in the 16-year-old NP-C patient compared with control subjects. The concentration of SNAG-∆^5^-CA and its metabolites was increased twofold in patients with 3b-HSD deficiency. The biochemical pathway for SNAG-∆^5^-CA formation is not yet clear. It appears that SNAG-∆^5^-CA is conjugated with GlcNAc at the C-7 residue and that, after exiting the hepatocytes, it is secreted in urine. Although the SNAG-∆^5^-CA cholesterol elimination pathway is not significant in healthy subjects, it has a potential to be a major biomarker for NP-C, although studies that would include much higher numbers of patients and controls are required to support this.

## Summary and suggested diagnostic approach

Technological advances in all areas of biomedical science have already led to novel treatment developments in many inherited metabolic diseases that have previously been considered untreatable. This progress will continue, and requires parallel improvements in clinical diagnoses. This is particularly important in neurodegenerative disorders where early detection and treatment may prevent or lessen irreversible damage. The worldwide availability of gene sequencing makes it the most important diagnostic test in NP-C, and recent developments in sequencing technology have reduced costs and improved turnaround times.

**Fig. 1:**
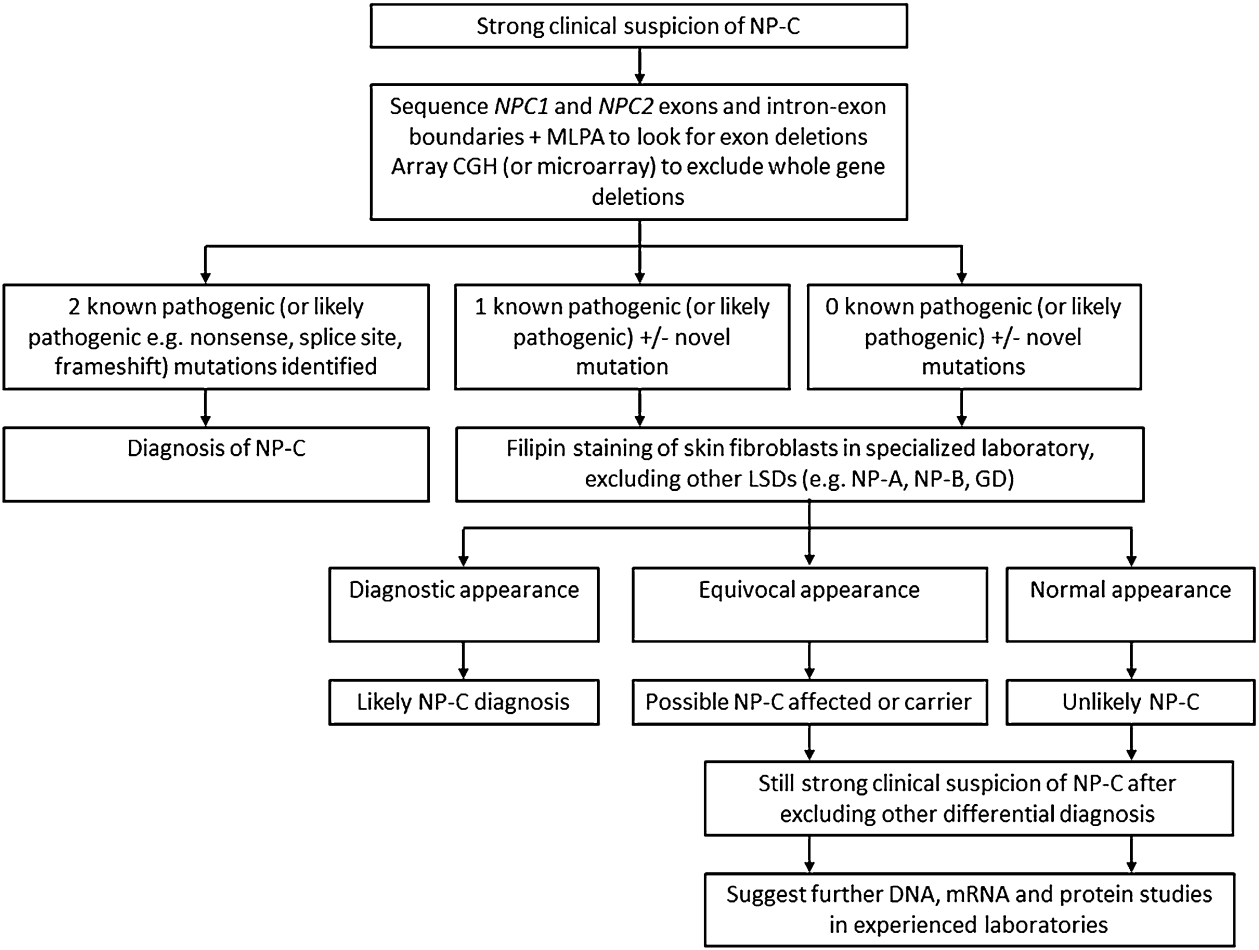
Proposed, simplified diagnostic algorithm for NP-C focusing on genetic mutation analysis.* GD* Gaucher disease,* LSDs* lysosomal storage disorders,* MLPA* multiplex ligation-dependent probe amplification,* NP-A/NP-B/NP-C* Niemann Pick disease types A, B and C

We suggest that patients with a suspicion of NP-C should undergo *NPC1* and *NPC2* gene sequencing as standard. Identification of two pathogenic mutations that segregate in the family should be sufficient for at least an initial diagnosis (Fig. 1). Further investigations in specialised laboratories are recommended for patients in whom only one pathogenic mutation is identified, and for those with novel mutations. These investigations may include molecular genetic approaches such as MLPA, RNA sequencing, quantitative real-time PCR and western blotting. Filipin staining of skin fibroblasts should also be undertaken bearing in mind the possibility of an equivocal result.

In the near future, it is expected that LC–MS/MS methods will become available and will complement molecular genetics, thus affording accurate, early and fast diagnosis in all patients with NP-C.
